# Paying to Normalize Life: Monetary and Psychosocial Costs of Realizing a Normal Life in the Context of Free Antiretroviral Therapy Services in Uganda

**DOI:** 10.1177/2325958219859654

**Published:** 2019-07-03

**Authors:** Esther Kalule Nanfuka, David Kyaddondo, Sarah N. Ssali, Narathius Asingwire

**Affiliations:** 1 Department of Social Work and Social Administration, Makerere University Kampala, Uganda; 2 School of Women and Gender Studies, Makerere University Kampala, Uganda

**Keywords:** antiretroviral therapy, money, normal life, people living with HIV, psychosocial costs, Uganda

## Abstract

Antiretroviral therapy (ART) is considered the treatment that enables people living with HIV (PLHIV) to lead a “normal life”. In spite of the availability of free treatment, patients in resource-poor settings may continue to incur additional costs to realize a normal and full life. This article describes the monetary expenses and psychosocial distress people on free ART bear to live normally. We conducted in-depth interviews with 50 PLHIV on ART. We found that the demands of treatment, poverty, stigma, and health-system constraints interplay to necessitate that PLHIV bear continuous monetary and psychosocial costs to realize local values that define normal life. In the context, access to free medicines is not sufficient to enable PLHIV in resource-poor settings to normalize life. Policy makers and providers should consider proactively complementing free ART with mechanisms that empower PLHIV economically, enhance their problem-solving capacities, and provide an enabling environment if the objective of normalizing life is to be achieved.

## Introduction

Seated on a wooden bench with two men at Naggalama Hospital, one of the authors waited to enter a counselling room. The two men were engaged in a conversation and did not seem bothered by the author’s presence, when a middle-aged woman wearing a long over-sized dress and holding a big sisal bag approached them. She greeted them casually and took out 2 packets of a brown powder from the sisal bag. The contents were visible through the colorless polythene package. Holding them up, she said, “These are avocado seeds. They are very good especially for us who take medicine [ARVs]. We suffer from blurred vision. Just pour some in your tea and drink.” One of the men enthusiastically asked for a packet. He appeared to have suddenly found a solution to his problem. He took the powder in his hands, examined it critically, turning it around several times before inquiring about the price. The woman told him that it would cost “only” 2000 Uganda shillings (UGX; note 1). He got up, reached for his trouser pocket, pulled out a creased 2000 UGX note and gave it to the woman. She thanked him and moved on to the other clients in the queue. When she left, the author turned to the man and inquired if the powder would help him. In a rather somber tone he replied, “*Huh, mwana wange obulamu tugula bugule*”. (Huh, my daughter, we have to pay for life).

“Paying for life” as revealed in the vignette above epitomizes the monetary and other economic and psychosocial costs that people living with HIV (PLHIV) on medicine have to bear in their day-to-day quest for a “normal and full life”.

**What Do We Already Know About the Topic?**

The rollout of free ART has enabled multitudes of people living with HIV in resource-poor settings to access life-prolonging antiretroviral medicines and lead a normal or close to normal life.

**How Does Your Research Contribute to the Field?**

It highlights the central role of money in enabling PLHIV on free ART to attain other key values in life like privacy and marriage other than good physical health. It further highlights the financial and psychosocial burden PLHIV in resource-poor settings endure to realize a normal and full life amid structural barriers.

**What Are Your Research Implications Toward Theory, Practice, and Policy?**

It underscores the urgency of strengthening the economic and psychosocial support components of free ART programs in resource-poor settings, if its objective of normalizing life is to be attained.

Rolling out free antiretroviral therapy (ART) has enabled multitudes of PLHIV in resource-limited settings to access life-saving antiretroviral (ARV) medicines and live longer. Uganda was one of the first countries in Africa to scale up free ART. Using resources from 3 global initiatives, the Global Fund for Tuberculosis, AIDS and Malaria, the US President’s Emergency Fund for AIDS Relief, and the Multi-Country AIDS Programme of the World Bank, the country embarked on accelerating the scale up of ART in 2004.^1^ It is indicated that ART was available to 1 028 909 PLHIV in Uganda by mid-2017, which constituted over 70% of the estimated HIV-infected population in the country.^2^

Antiretroviral therapy is considered the treatment that enables PLHIV to manage their condition and lead a “normal life”.^3^ However, evidence suggests that PLHIV on free ART in resource-limited settings may find it difficult to realize the benefits of the treatment without investing money, enduring distressing situations and sometimes compromising personal convenience, interests, and well-being (trade-offs). For instance, in Uganda and several other sub-Saharan African countries, PLHIV on ART are encouraged to adhere to not only HIV medicines but the broader aspects of the treatment regime for better outcomes. These include nutritional requirements, regular attendance of ART clinics for review and refill (pick new supplies of medicine), and prompt treatment of opportunistic infections (OIs), side effects of ARV medicines and any other illnesses, among others.^1,3–5^

Previous studies show that even while on free treatment structural barriers such as poverty and health system limitations necessitate that PLHIV in sub-Saharan Africa continue to invest money, endure distress, and compromise personal convenience to be able to adhere to the broad ART regime. A study of adherence in Uganda found that some food insecure patients only managed to adhere to medication by sometimes taking it on empty stomachs and enduring distressing side effects.^6^ In South Africa, Chimbindi et al found that PLHIV on ART require substantial amounts of time and money to travel to the health facility for refill.^7^ In Nigeria, Uganda, and Tanzania, financial constraints often compelled PLHIV to forego basic needs such as food to save money for traveling to the health facility for refill.^8^ Therefore, besides the provision of free treatment, PLHIV in resource-poor settings may require broader socioeconomic interventions to enable them realize the normal life that ART promises them.

## 

### What Is Life?

Life (*obulamu*), as used among the Baganda of central Uganda, is a broad and encompassing concept. It depicts both the state of the body and social experience. A person who is critically ill, on a deathbed, or frail is described as *talina bulamu* (literally lifeless) or *obulamu butono* (little life). In addition, *obulamu* may also be used to refer to the state of being alive, dead, sick, or healthy. The derivative word, *mulamu*, means alive or free from an infection or disease as opposed to being sick/infected or dead. In this regard, HIV-positive people are commonly identified and sometimes identify themselves as *balwadde* (sick people) or *mulwadde* (singular). Their uninfected children or spouses are referred to as *mulamu*, which in this context means not infected. These expressions underline the intimacy between health and life in the local moral worlds of the Baganda. Good health is conceived as an integral component of life,^9^ and in some cases (eg, in the story at the beginning of the article), the term *obulamu* refers to good health. However, in practice, *obulamu* incorporates broader dimensions of social experience.^10^ It also describes feelings. *Kulya bulamu* means having fun, while *mpulila obulamu* denotes feeling well/relief especially following a difficult situation. *Obulamu* is also used to capture an individual or people’s lived experience. Baganda and other Bantu tribes in Uganda often inquire about the others’ lived experience (*obulamu buli butya?* How is life?) after exchanging greetings. The inquiry may be narrowed down to a specific aspect of one’s life experience such as work (*obulamu kumulimu*—life at work).

For the lay person in Uganda, life principally revolves around keeping alive, attaining and sustaining good health, work/livelihood, economic security, social relationships, marriage, bearing children, personal image, feelings of self-worth, satisfaction and control, affirming status and power, pursuing aspirations, and fulfilling life tasks (instituted social roles and obligations).^9,11,12^ Such values define a “normal” and full life in this setting. Thus, by normal life, we mean the realization of broader values and goals that characterize “normal living” in the sociocultural context of Uganda.

The costs attendant to realizing a “normal life” while on ART have had limited focus in most HIV/AIDS studies, despite the notable interest of several scholars of sub-Saharan Africa in the lives of PLHIV on medicine. Existing studies mainly show how HIV medicines have enabled HIV-positive patients to regain health and return to the normal struggles, concerns, and aspirations that characterize living in their sociocultural contexts (normalization of life), but provide no rigorous accounts of the economic and other burdens involved.^11,13^ The article seeks to contribute to an understanding of the economic and psychosocial costs of normalizing life among PLHIV on free ART in a resource-limited setting. It further seeks to highlight the underlying structural constraints that render it difficult for PLHIV on ART in a resource-poor setting to realize key life goals without investing money or enduring distress. Therefore, 2 main research questions are addressed: what economic and psychosocial costs do PLHIV on ART bear to live a normal and full life? and what structural constraints render it difficult for PLHIV on ART in a resource-limited setting to normalize life without bearing economic and psychosocial costs? The article explores how ART in a context of scarcity and stigma necessitates PLHIV on treatment to incur interminable economic (money and time) and psychosocial costs to achieve and sustain values that pertain to a normal and full life, particularly marriage and intimacy, privacy, and good health. By psychosocial costs, we mean distress and trade-offs associated with an interplay of patients’ emotional or mental states and their response to social situations. We argue that normalization of life for PLHIV requires more than providing access to free HIV medicines. It necessitates a holistic approach that purposely and substantially integrates medical, economic, and psychosocial interventions.

## Methods

### Study Design, Setting, and Population

This article draws on data from an ethnographic study that sought to examine how PLHIV on medicine in Uganda mobilize resources for managing HIV. The ethnographic approach enabled generation of a rich and nuanced understanding of the lived experiences of PLHIV within their contexts.^14^

The study population included adult males and females aged 18 years and above enrolled on the ART programs of Naggalama Hospital and Mukono Health Centre IV (HCIV). Both facilities are situated in Mukono district, a central peri-urban area. Naggalama Hospital is a mission/private not-for-profit health facility located in the rural part of Mukono, while Mukono HCIV is a public health facility in the heart of Mukono Town. Respondents were selected from the 2 health facilities on the premise that the differences with regard to the location (rural versus urban) and management (mission versus public) would provide deeper insights into the varied experiences of clients. For instance, mission establishments, unlike public facilities, usually charge user fees, a practice that has different resource implications on the respective clients.

Naggalama Hospital and Mukono HCIV are the largest health facilities in Mukono district. Both facilities attract patients from the neighboring districts, including Kampala City, which is situated about 20 km from Mukono district headquarters.^15^ In September 2015, records of the 2 facilities indicated that Naggalama Hospital had 2862 active clients, 66.4% of whom were female, while Mukono HCIV had an estimated 3500 clients, of whom 68.1% were female. The most recent National Population and Housing Census estimates the population of Mukono district at 596 804 people.^16^ Available reports show that 7% of the district’s population is infected with HIV.^15^

### Sampling

The study was conducted in 2 phases: the initial phase covered 50 respondents (25 from either health facility) who were purposively selected to capture variations among PLHIV on ART. The participants were selected on the basis of gender, marital status, age, and length of period on ART. In addition, the study considered whether a respondent was only on prophylaxis or on prophylaxis and ARV medicines, disclosure status, and presence of any illness. The selection criteria allowed a mix of both demographic and treatment-related aspects, to provide insight into the diverse experiences of PLHIV on ART. Eligible participants were identified from the register on clinic days and approached for interviews by the researcher between September and October 2015. After identification and sampling from the register, heath workers were helpful in getting us in contact with the participants by locating them among the many clients at the clinics. The potential participants were approached after receiving HIV care. The researcher explained the purpose of the study to the selected participants. They were informed that there were no immediate benefits (monetary and otherwise) from their participation, but the information obtained would provide insights into the experiences of PLHIV on ART as they mobilize treatment resources, that could be used by policy makers to address any issues of concern. They were further informed of their right to nonparticipation and how refusal to participate in the study would not in any way affect their access to services from the health facility. The selected participants were required to accept or decline to participate on the spot. A total of 67 PLHIV were approached for interviews before the target sample of 50 was generated. The 17 who declined to participate gave various reasons including having no time due to busy work schedules, feeling worn-out after waiting for long at the clinic, being noninterested, and having urgent matters to attend to at home. During the second phase, 15 of the 50 participants were selected for continuous follow-up to get deeper insights into their day-to-day illness experiences. The article draws on both the experiences of the 50 respondents and others encountered during participant observation at the 2 facilities.

### Data Collection

The 50 respondents were involved in in-depth interviews with the aid of a guide consisting of open-ended questions. Initial contact with participants was at the health facility. Each interview lasted approximately one and a half hours and explored participants’ illness history, conceptions of the resources that are necessary for the management of HIV and how they mobilize them, and constraints and opportunities that they encounter in mobilizing resources. Participant observation was conducted at the 2 facilities for 6 months between September 2015 and February 2016. One of the authors attended and participated in the activities of 2 out of the 3 clinic days offered by each of the 2 health facilities where participants were contacted. At the health facilities, the author attended counseling sessions and health education talks, after consent of both patients and health workers, and sometimes assisted in counting medicines. It is common practice for ART providers in Uganda to request patients and other people (such as community volunteers) for support in counting medicines, given the human resource shortages in most of the facilities. The author also often interacted with and participated in clients’ conversations. Through participant observation, we were able to capture actions as they occurred in a natural setting.^17^ Participant observation further facilitated continuous engagement and interaction with health workers to understand the organization, procedures, requirements, and context of HIV treatment and care. The mix of methods enabled triangulation of data. The interviews were audio recorded, while observations were documented in field diaries on a daily basis.

### Data Management and Analysis

The audio-recorded interviews were transferred to a lockable computer for storage, to minimize access by unauthorized individuals. The interviews were transcribed verbatim and translated into English with the assistance of language experts. Three of the authors participated in transcribing and translating some of the interviews. The audio interviews were destroyed after transcription. The field notes were word-processed by the authors. Both interview transcripts and processed field notes were stored on computers with passwords to limit access to only the authors.

The processed interviews and field notes were imported into NVIvo.10 qualitative data analysis software package for further management. The analysis process involved reading and rereading the data as sentences, and paragraphs and whole sections were coded according to the identified themes and categories. In generating themes for the development of this article, both deductive and inductive thematic analysis were conducted.^18^ We started off with a broad theme “paying for life,” which was derived from the data as indicated in the vignette at the beginning of the article. This theme was defined as any monetary or other costs incurred by patients in their quest to attain/maintain good physical health. Categories under this theme included costs associated with ART adherence and other treatment-related costs. Data coded under the former category included costs associated with regular travel to the health facility for refill such as transport, expenditure on treatment of OIs, and patients’ complaints about the burden of taking the daily pill.

Further scrutiny of data revealed that PLHIV valued and worked to attain more than good health. They also sought privacy and getting and keeping good intimate partners, among other things. An interrogation of the concept life as used in the local Ugandan context revealed that seeking for privacy and intimacy are part of peoples’ day-to-day social experiences and thus can be seen as integral components of PLHIV’s life in its broad sense. We identified the concept “normal life” from mainly anthropological literature on the treatment experiences of PLHIV on ART and interrogated it in light of how life was defined in the local Ugandan context. Our aim was to identify values and goals that constituted what was considered a normal and fulfilling life in this setting. We found that good health, privacy, and intimacy were some of the goals that people aspired to attain.

**Figure 1.**
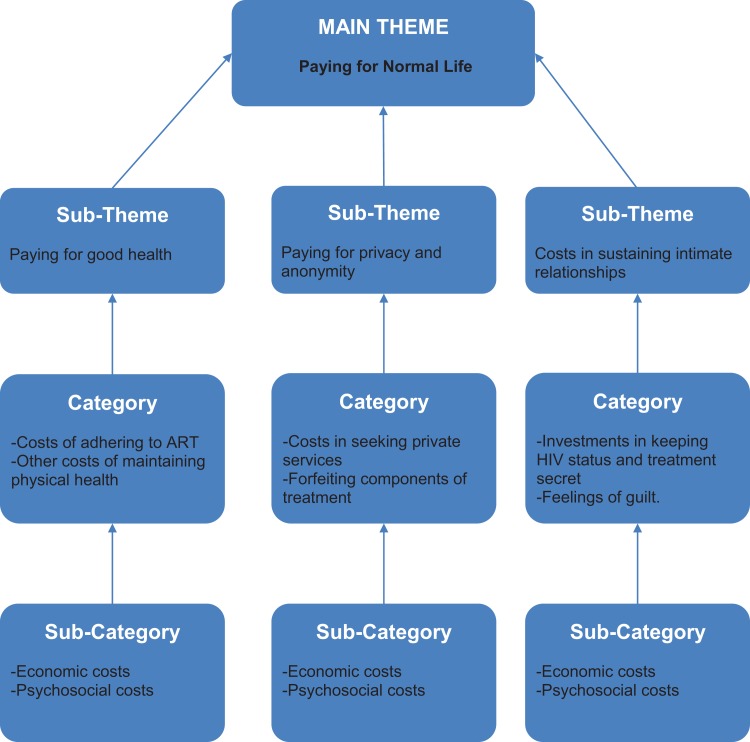
A summary of themes and subthemes.

These revelations informed the revision of the main theme “paying for life” to “paying for a normal life”. The meaning of the theme was broadened to include sacrifices and economic, social, and psychological costs that PLHIV on ART endure to achieve the states and goals that define normal living in the sociocultural context of Uganda. Categories under the original theme were collapsed into a subtheme “paying for good health.” Two other subthemes “paying for privacy and anonymity” and “costs in sustaining intimate relationships” were created. Data under each subtheme were further reread and categorized into the nature of costs involved. Drawing on our knowledge of various costs, we categorized data into economic, social, and psychological costs. Data under social and psychological costs were eventually collapsed into a broader category psychosocial costs. In the process of reading and coding the data, cases of PLHIV who failed to adhere and maintain privacy or intimate relationships due to lack of financial capacity or unwillingness to make sacrifices were identified. These were coded under the category “failure to meet life goals” to ease data retrieval for comparison. The themes and subthemes on which the article is based are summarized in Figure 1.

### Ethical Approval and Informed Consent

The study was granted ethical clearance by Mengo Hospital Research Ethics Committee (679/4-15) and Uganda National Council for Science and Technology (SS 3939). Written informed consent was obtained from the 50 participants selected for the study. Respondents who could not write used a thumbprint. Verbal consent was obtained from all the 15 clients selected for follow up. Their acceptance to participate in the second phase of the study was recorded on the original consent form they had signed. The information shared and observations made were kept confidential. At the health facility, the clients were informed that the author was a researcher. For purposes of observing confidentiality, only pseudonyms are used in the article.

## Results

### Characteristics of Study Participants

Thirty-one of the 50 PLHIV were female. Ten of the participants were on only co-trimoxazole or its substitute, dapsone prophylaxis, because their CD4 counts were still above the threshold for initiation on ARV medicines at the time. Their period on ART ranged from being newly initiated to 9 years. However, we found no significant variations in the experiences of participants by medication and treatment duration.

In terms of age, the majority (41) were in the reproductive age-group of between 18 and 49 years. The majority (33) of the participants were married or cohabiting. Eleven of the married respondents had not disclosed to their spouses. In terms of occupation, the majority (26) mainly depended on farming for a livelihood (see Table 1).

**Table 1.** Characteristics of Study Participants.

Characteristic	Frequency (N = 50)	Percentage (%)
Medication		
ARV medicines and prophylaxis	40	80.0
Only prophylaxis	10	20.0
Gender		
Male	19	38.0
Female	31	62.0
Marital status		
Single	5	10.0
Married/cohabiting	33	66.0
Divorced/separated	8	16.0
Widowed	4	8.0
Main source of livelihood		
Farming	26	52.0
Salaried employment	5	10.0
Petty trade	4	8.0
Casual labor	3	6.0
* Bodaboda* (motorcycle taxi) riding	3	6.0
Artisanary/mechanics	2	4.0
Nothing	7	14.0
Age, years		
Below 20	2	4.0
20-29	13	26.0
30-39	15	30.0
40-49	11	22.0
50-59	6	12.0
60+	3	6.0
Treatment duration, years		
Below 1 year	5	10.0
1-4	27	54.0
5-9	18	36.0

Abbreviation: ARV, antiretrovirals.

In the following sections, we present data on the psychosocial and economic costs and underlying structural constraints to realizing the values of good health, privacy, and marriage among PLHIV enrolled on treatment. These data are presented under 3 themes: the cost of attaining and sustaining good health, paying for privacy and anonymity, and secrecy and guilt in intimate relationships.

### The Cost of Attaining and Sustaining Good Health

During participant observation at the 2 facilities, we often heard health workers informing PLHIV that initiating ART would control HIV disease progression and improve or sustain their health. However, they always emphasized that optimizing the health benefits of treatment was contingent on adhering to HIV medicines, eating a balanced diet, visiting treatment centers regularly (on a quarterly basis for our respondents) for refill and clinical review, avoiding worries and strenuous activities, and managing comorbidities promptly.

“Eat well, avoid worries and strenuous activities, [and] take your medicines daily; you will be well,” were catchwords in HIV counseling sessions orientating clients commencing ARV medicines. We found that for several of our interlocutors, complying with the “rules” of ART involved a number of psychosocial and economic costs including those related to transport, food, and medicines as described below.

#### Transport—A necessary cost

The ability to make the required routine journeys to the treatment center is a critical step in accessing and maintaining a constant supply of the life-giving HIV medicines. We found that most of our interlocutors required money to facilitate regular travels to the treatment center for refill. Some patients required money for transport because they lived far from the nearest treatment center as Micheal who lived about 50 km away explained.

A sick [HIV positive] person should have money. Now like me I have needs…Then transport. It is a big problem. Like me who comes from far. I need money to travel here [treatment center]. I first get a *bodaboda* (motorcycle taxi) to take me to the highway. That one charges me UGX 10 000. From there I board a taxi up to here [treatment center]. That one charges me UGX 3000. You see I spend a lot of money on transport.

Other clients preferred to seek care from distant health facilities, even when HIV treatment was available at a closer facility. For some patients, seeking care from distant facilities was a way of maintaining anonymity and averting potential stigma from close relations. Asked why she decided to enroll at a facility over 70 km away instead of closer facilities in her home district, Gauda a middle-aged businessperson explained:

My friend, it is difficult [*to explain*]. I have not disclosed to my husband that I am on medicine [ARVs]. I learnt that I have HIV during the pregnancy of my second child. I thought about it and decided not to inform my husband. I feared that he might leave me…I decided to seek treatment from here because I know most people do not know me. I am at least confident that no one here can inform my husband that I am on medicine.

While seeking care at a distant health facility enabled Gauda to keep her HIV status anonymous, she expended considerable amounts of money on transport and regularly risked her life traveling far in the wee hours of the morning to make it to the treatment center early enough.

I use about 25 000 (US$6.8) on every visit. This includes money for hiring *boda-boda* up to the highway [and] eating something when I arrive here. You know, I wake up very early, at 4: 00 am I am on the road. So I have no time to eat before leaving home. I have to get here early enough to be among the first people to be seen [by the Clinical Officer]. You see the line; I cannot afford to stay here for long. I have a shop to attend to.

Unlike Gauda, some clients chose a distant health facility mainly because they perceived it to offer superior treatment services. Ben, who was open about living with HIV, traveled over 30 km to Naggalama, instead of seeking HIV care from a nearby HCIV.

I live a bit far, about 35 km from here, but prefer coming here because I know this facility is well equipped. They may tell you to do a scan or an X-ray. These services are not available at the HCIV. You would have to go to another facility. But here at Naggalama most services are available.

For Penina, the decision to travel over 170 km from where she was employed to Naggalama Hospital was reportedly motivated by the need to safeguard her slot at this facility.

I keep coming to Naggalama [Hospital] because I do not want my slot to be given to another person. I am in my current place of residence because of work; I can lose the job anytime. My parents’ home is not very far from here. I can’t change. I hope to return to my parents in case I get very sick or when the job is no longer available.

Penina’s actions were informed by the peculiar modalities that define clientship in mass ART programs within Uganda. While patients with other illnesses are free to access health care at their convenience, PLHIV on ART are required to receive HIV treatment services only where they are registered. Once a transfer is sought, it is considered permanent; it is difficult to retract it or renew clientship at the initial facility. Health workers reported that the strict procedures were implemented to discourage unnecessary requests for transfers and the potential confusion that could ensue when clients frequently changed treatment centers. However, we found that the procedures often push clients who live in uncertainty over future prospects to cling onto slots at distant health facilities at a huge cost. For instance, Penina not only spent a lot of money (about UGX 30 000; US$8.1) to travel to and from Naggalama Hospital to her place of work routinely, but she also lost productive time as she usually traveled a day before and spent the night at her natal home to be able to make it to the treatment center in time.

The requirement of routine clinic visits in a context of stigma, long distances to health facilities, ill equipped facilities, and peculiar modalities of clientship implemented by ART programs often renders it inevitable for PLHIV to incur financial, temporal, and opportunity costs in accessing free HIV treatment services.

#### Food and the cost of taking the daily pill

Health workers identified regular access to food as critical to thriving on treatment and taking the daily pill. In the counseling sessions we attended, health workers emphasized to PLHIV that they should take their medicines daily and make an effort to eat a sufficient and balanced diet consisting foods rich in carbohydrates, proteins, and vitamins to improve their efficacy. Clients were further advised to preferably take their medicines after or immediately before a meal to minimize the occurrence of possible side effects.

All our interlocutors reported that they needed money to meet the nutritional requirements of the treatment. Most of them indicated that they mainly fed on own-grown food, but had to supplement it with that from the market to balance the diet. Sperito, a pensioner in his early 80s related, “I have a farm where I grow fruits, *matooke*, sweet potatoes, beans, and maize. But as you can see, that food is not balanced enough. We supplement it with meat, milk, and vegetables; those ones I buy.”

Namugerwa, a widow in her early 50s, reported, “Many of us cannot get all the foods unless we buy. We mainly grow sweet potatoes. To balance the diet, I normally sell some of them and get other foods.”

The necessity of money in accessing an adequate quantity and quality of food as required was further illustrated by incidences of crop failure as recounted by Mugwanya, a prominent farmer.

I mainly eat *matooke* (green plantain). I have a plantation at home, but in January the heat was too much, then most of my banana plants withered. I now rely on trucks that bring *matooke* from as far as Mbarara [a big town in southwestern Uganda]. The beans dried due to the heat. I did not harvest anything. I just buy…

Other patients observed that the expenditure on food was often triggered by the need to manage side effects of ARV medicines.

They [PLHIV] can really eat. When you start medicine [ARVS], uumh…you can eat. I started the medicine when I was light, but started eating a lot [of food]; I even added some kilos. Sometimes I didn’t have much to eat, but I had to look for it; from the garden and when possible I would buy. My appetite was big; the body was demanding. (Badru, a male participant)

These days I crave a lot. It must be the medicine [ARVs], [because] I never used to be like that. Most times I don’t feel like eating our usual food. I usually desire to eat soft food like matooke, pork, chicken, fish, and when I don’t get them it is a problem. They say it is pregnant women who crave, but ha! [my dear] a person on [HIV] medicine also craves. Sometimes, the body yearns for chicken. You feel a sense of urgency and become disturbed. You only get better after eating what you are craving for. That is where problems emerge; that is why you need to have money. When “the heart says” [you yearn for] chicken or pork, you buy and eat to stop the yearning. Otherwise, you remain distressed. (Yiga, a male participant)

Some of the patients who could not obtain a regular supply of food due to financial constraints reported enduring distressing side effects to adhere to medication. An example was Rita, who reported that she experienced side effects when she took her medicine without eating, but continued to do so even when food was unavailable.

I get extremely dizzy when I take this [ARV] medicine without eating. The head feels heavy, akin to that of a drunk person. But I have no option, even when I have no food, I take it. Medicine is my life. I cannot leave it.

Besides, taking the daily pill takes considerable dedication and endurance from the patient. Several of our interlocutors complained about the inconvenience of taking daily pills; some of them wished for injections. A female client encountered during participant observation at one of the facilities described her situation as thus:

I have always hated tablets. Their taste makes me sick. I wish there were injections; I would have quickly opted for them. Taking this medicine on a daily basis is difficult for me. I have had to invent a way of swallowing without tasting it. I roll each tablet in a lump of food and swallow, then follow it with water. Of course sometimes I get choked, but what can I do, I want to stay alive.

Clients who were not as resolved to continue on HIV medicine amid constraints including serious side effects commonly abandoned the drugs. As we participated in the activities of an ART clinic at one of the facilities, we observed an elderly client walking straight to the dispensing window, dumping there a black polythene bag containing his newly initiated ARV medicines and saying to the nurse:

Here is your medicine. I do not want it anymore. I have taken alcohol all of my life, but I have never felt this drunk. How can one sleep and wake up drunk for a whole week? Can you imagine I stopped taking this medicine a week ago but I still feel dizzy? Take it, I am no longer interested, I would rather die than stay drunk for the rest of my life.

As shown, the lack of self-sufficiency in food production, incidences of crop failure, side effects of ARV medicines, and poverty may render adherence to the daily pill and/or complying with dietary recommendations to attain and sustain good health difficult without money or endurance of distressing situations.

#### The inevitability of buying medicines

Stock-outs of essential drugs including ARV medicines and co-trimoxazole/dapsone prophylaxis remain common in Ugandan health facilities.^19^ Therefore, adherence to ART often necessitates that clients use own money to buy medicines that are out of stock if they are to minimize interruptions in their treatment. This was attested during participant observation at Mukono HCIV in October 2015, when administrative challenges in the procurement unit of the facility led to a stock-out of both co-trimoxazole and an ARV regimen called Combivar (a combination of zidovudine [AZT] and lamivudine [3TC]) for 2 weeks. Clients on only co-trimoxazole were advised to provide for themselves a monthly dose and return after a month. Those on combinations with Combivar were told to buy it and received the drug that was available. Conversations with clients that had been affected by the stock-out of Combivar and returned for refill 2 weeks later, as advised, revealed that several of them had failed to obtain the drug due to financial constraints. However, those who could afford reported that they had paid between UGX 65 000 and UGX 70 000 (US$17.6-18.9) for a tin. According to the ART Policy, mass treatment programs do not sell ARV medicines, but they are available among commercial providers.^20^

For clients taking dapsone, an expensive drug given to patients allergic to co-trimoxazole, buying medicine was the norm. Bwire whose wife Betina took Dapsone reported, “The truth is I always buy her medicine, it is always out of stock. We have found it there only about 5 times for the 6 years we have been clients at that treatment center.”

Similarly, Veronica told of how health workers rationed the few available doses of dapsone, necessitating her to buy the rest of the medicine.

It is tough being on dapsone. While my fellow patients on co-trimoxazole are given drugs for the entire 3 months, I am normally given medicine for only 1 month. I always have to look for money to buy medicines for the 2 months…

Besides ARV medicines and prophylaxis, ART clients often required money to buy drugs to manage other illnesses. These included OIs, the serious side effects of ARV medicines, and common illnesses including malaria. The Uganda ART Policy^20^ requires mass treatment programs to provide free medicines for treating other ailments as part of comprehensive HIV care. However, compared to ARV medicines and prophylaxis, the supply of medicines for the management of comorbidities (like antibiotics and antifungals) was more irregular. We observed that several of our interlocutors were advised to buy the medicines prescribed for the management of OIs and other ailments because they were out of stock. It was not uncommon for them to fail to buy the prescribed medicines due to lack of money. An example was Sikola who failed to buy medicines prescribed for treating a urinary tract infection (UTI) and palpitations for 6 months. She explained;

I haven’t been well for a while. The health workers advised me to buy some medicine for managing an UTI and palpitations about 6 months ago, but I am yet to get money [to buy it]. The last time I came [to the treatment center] I thought I would find the medicine but it was not there.

Like Sikola, we observed that several patients we encountered during participant observation in the 2 health facilities returned for refill without purchasing the medicines they had been prescribed to treat illnesses reported on the previous visits.

In addition, our interlocutors with persistent illnesses commonly spent on a cocktail of herbal medicines in the quest for a cure to their ailments, which further increased the financial burden of treatment. Kagoya who had lived with a skin rash for about 8 years described the cocktail of medicines and herbal remedies she had used to try and cure the condition.

They have given us all sorts of tubes [ointments] for smearing and medicines for taking but the skin has refused. Sometimes they write for us tubes to buy, when they are not available at the health facility…Recently, I was going to dig then I found a car with a loud speaker advertising herbal medicines for cough, skin rashes and others; then I bought a tin for the skin at UGX 5000 (U$1.35); but it didn’t work. I have also used skin guard (a type of medicated soap); someone recommended it to me. A friend told me to use a salt block for cows. I have tried it several times, but didn’t see significant improvement in the skin…These days I buy Vaseline and mix it with warm salty water, then smear on my skin, a friend told me it would help…

The client in the vignette at the beginning of the article also reported simultaneously using bio and herbal medicines to cure blurred vision for over a year to no avail.

I get blurred vision every now and then. I have tried everything to cure it. When they give me medicine here [Naggalama Hospital], I use it. But I also try virtually everything I am told can cure the condition. However, it has persisted. It is over a year now.

The despair to improve his health, reflected in the excerpt above, could explain the enthusiasm with which this client bought the avocado powder that was marketed as a solution to the condition.

Essentially, stock-outs and ineffective management of illnesses from the health facility may render it inevitable for PLHIV to buy medicines in an attempt to improve and sustain good health.

### Paying for Privacy and Anonymity

Privacy is one of the valued ends that HIV care in Uganda seeks to uphold by promoting the principle of confidentiality. However, the setting and arrangement of ART clinics often makes it difficult to uphold the privacy and anonymity of clients, at least concerning their HIV diagnosis.

We found that HIV treatment at the 2 facilities was provided by isolated units known as ART clinics. The clinics operated on designated days and attended to only HIV-positive patients. Clients typically waited in an open space where a desk for conducting triage (registration, measurement of weight, blood pressure, temperature, nutrition status, and height) was placed. The clients’ names were called out one at a time for triage, before they queued to see a clinician. While the arrangement facilitated the management of large numbers of patients, the open setting made it difficult for them to keep their status secret. Sperito told of how he discovered the HIV status of a village-mate, who preferred to remain anonymous from the ART clinic.

…There is a man from my Village I found here at the health facility. When I asked him why he was here, he claimed that he had a patient admitted in the wards. The annoying thing is that later his name was called at the triage while I was seated in the waiting area, then I realized that he had lied to me…

On our second visit to Carol, who had just relocated to a new town, she narrated how her efforts to keep her HIV status secret had been recently undermined by the lack of privacy at ART clinics.

Like I told you, I decided to get medicine from Naggalama [Hospital] instead of Mukono, even though it is near, because many people in Mukono [Town] have known me since childhood. But the last time I was there [Naggalama Hospital] my former neighbour saw me. I did not see her, but when I went back home she came to my house and told me that she had seen me at Naggalama. She said that she was seated in the waiting area when she saw me placing my book on the table [at the triage]. I was alarmed. I immediately knew that my secret was out. I thought that the right thing to do was to relocate to another place. That is why I am living here now.

During participant observation at the 2 clinics, we observed that privacy was also compromised in the process of triage. Health workers usually gave patients preliminary advice on adherence, hygiene, nutrition, and other identified health issues during triage. Some of the issues discussed were sensitive and personal. Since triage desks were placed in the open and close to large groups of patients often engaged in conversation, the health workers usually projected their voices to be heard and sometimes ended up embarrassing the clients. Yowanina was lectured about hygiene when she presented a treatment book that was soiled with mud to the nurse handling triage. Disappointed, the nurse raised her voice and inevitably attracted the attention of patients in the waiting area. Some started stretching their necks, while others moved closer to see the person in trouble. Smart in a sparkling white *gomesi* (traditional gown for women in Buganda and several parts of Uganda) fastened with a blue sash, Yowanina implored the nurse to lower her voice but several people in the crowd had already heard. She felt so embarrassed that she took refuge in the more deserted rearmost part of the health facility.

To avoid such exposure, some patients paid for private services. Naggalama Hospital had a well-instituted arrangement for clients seeking privacy and/or a quick service. Private clients did not attend the general clinic, but walked straight to a secluded room where a designated doctor and nurse received and attended to them. The nurse took all the necessary measurements and picked the clients’ medicines from the pharmacy. However, these clients paid 5 times more – UGX 10 000 (US$2.7) in comparison to a nominal fee of UGX 2000 (US$0.5) that the general clients were charged. Mukono HCIV had no instituted arrangements for providing private services. Being a public facility, clients were not charged any money to access HIV treatment. However, patients seeking privacy and anonymity usually avoided visiting the treatment center and paid health workers informal fees ranging from UGX 3000 to UGX 5000 (US$0.8-1.4) to pick their medicines for them. By not visiting the ART clinic, these clients forfeited clinical review and monitoring tests, which are an integral part of HIV treatment. Thus, in attaining privacy and anonymity, they not only spent money that was not required to access HIV care but also sacrificed important components of the treatment, thereby putting their health at risk.

### Secrecy and Guilt in Intimate Relationships

Intimacy and marriage (note 2) in particular are key values that most people in Uganda and other parts of sub-Saharan Africa aspire to achieve. However, due to the fear of HIV-related stigma, several of our interlocutors realized intimacy at the cost of psychologically distressing acts of secrecy and feelings of guilt.

An example was Zerida, a woman in her early 30s. She was already initiated on ARV medicines when she married her current husband but chose not to disclose her HIV status to him. She did not know his HIV status but was worried that he could leave her if he learnt that she was positive; yet, she was not ready to end the relationship. She related, “I had no way of telling him [about my HIV status]. I was worried that he could leave me. Where do you find a man who accepts you with all your 4 children these days? He is really caring.”

We found that this marriage meant a lot to her and her children’s (from an earlier relationship) social and economic security. She marveled at how her husband had started her a vibrant business and provided for her children like his own.

I am not complaining. I get everything I want. The business my husband started for me is lucrative. Even getting money to come here is not a problem…That man has taken care of my children like his own. He pays their school fees. I met him when the youngest was in primary, now she is in senior 4.

Zerida was determined to prevent her HIV infection from standing in the way of a marriage that she treasured and took measures to hide her status from her husband. She had been receiving ART from a HCIII, about 20 km from where they lived but decided to move farther away to Mukono HCIV, a distance of over 50 km. Here, she least expected to find people who could recognize and inform her husband about her positive HIV status. She related, “I thought I would find nobody who knows me here, to spread information about my status; and so far so good. I have been coming here for a long time, but have never bumped into anyone I know.”

The change of treatment center meant she spent over UGX 10 000 (US$2.7) on transport for every visit, when she could access similar and perhaps superior ART services from a nearby hospital without incurring any transport costs. She went ahead to conceal her ARV medicines and to disguise them by transferring them from containers to polythene packs that are commonly used for packaging other drugs in health facilities within Uganda. The repackaging was aimed at averting suspicion in case her husband accidentally found the ARV medicines.

When I return home, the first thing I do is to remove my medicines from the tins to polythene packs, then I throw the tins in the latrine. My husband is not the type of person who looks everywhere, but you never know. When the medicine is in the usual polythene packs, even if he finds it he won’t become suspicious because it looks like any other medicine.

The ARV medicines are exceptionally packaged in special containers, which distinguish them from other pills commonly prescribed in Ugandan health facilities. The latter is normally put in paper or polythene packages. Possession of pills packaged in containers can therefore arouse suspicion of one’s HIV infection.

The secrecy enabled Zerida to protect her marriage from discord related to her HIV infection for 7 years by the time of this study. She nevertheless had to incur considerable financial, temporal, and psychosocial costs to hide her status (described above); she also endured feelings of guilt and insecurity about the relationship. Her fears and guilt were portrayed during our first interview. She narrated her illness experience with poise until when we inquired about the whereabouts of her husband. Appearing wary that we could inform him about her status, Zerida panicked and quickly acted to mitigate the situation. As tears rolled down her cheeks, she knelt and implored us not to inform her husband. “Don’t tell him, don’t tell him…he will leave me,” she pleaded.

## Discussion

The article has presented the economic and psychosocial costs PLHIV on ART in a resource-limited setting commonly have to bear to realize a normal and full life in the context of free treatment. The aim was to provide insights into the burden of and constraints to realizing the core benefit of ART (a normal life) in a context of scarcity, so as to underline the need for more holistic interventions that proactively and substantially combine medical, economic, and psychosocial interventions.

We have demonstrated that the stringent ART regime, in a context of endemic structural barriers such as health system limitations, widespread HIV-related stigma, and generalized poverty, creates demands and constraints that PLHIV on ART have to manage if a life considered normal in their local setting is to be attained. In this context, their efforts to normalize life take considerable amounts of resources including money and endurance of distressing situations. The interminable economic and psychosocial costs PLHIV on medicine have to bear in order to adhere and attain/sustain good health and other values in life like privacy, marriage, and intimacy reify the notion of paying to normalize life.

The ART prescribes a social and medical code^1^ that introduces new demands and costs in the everyday life of its clients. Following the ART regime in a resource-poor setting, ridden with stigma is difficult without money, endurance, and trade-offs. As Chimbindi et al show, adherence to medicines usually necessitates that PLHIV spend substantial amounts of time and money on routine transport to and from the treatment center for refill.^7^ These financial and temporal implications of accessing free HIV treatment are also discussed elsewhere.^21-25^ The cost is amplified for clients who feel compelled to travel to distant health facilities to avert potential stigma, save intimate relationships, keep their slots, or access better quality services. The tendency of PLHIV to opt for distant treatment centers for fear of stigma and to access services that are perceived to be of better quality has been documented elsewhere.^4,26^ The PLHIV also require money to guarantee lifelong compliance with dietary recommendations, even in a predominantly agricultural economy. This lends itself to what Whyte and Whyte observe, that self-sufficiency in the production of food is hard to achieve even in a predominantly agricultural economy like Uganda.^27^

Furthermore, it is common knowledge that HIV medicines may exacerbate hunger, big appetites^1,6,27,28^ and, as we found in this study, cravings, all of which increase the patients’ demand for food and often money to satisfy them.^29^ Adherence to medicines further necessitates that clients are able to provide for themselves medicines when free drugs get out of stock from treatment centers and that they are dedicated enough to bear the inconvenience of taking the daily pill for life. In addition, limitations in available treatment for OIs and side effects of ARV medicines often compel patients to simultaneously use herbs, other complementary and alternative therapies, and biomedicine in the quest for a solution. Using herbs while on ART not only increases the financial burden of managing the conditions but also puts the patients’ health at risk.^7,30^ The tendency of PLHIV on ART to combine biomedicine with herbs in the treatment of severe and/or persistent illnesses has also been reported elsewhere.^31^ Like Weiser and colleagues also found, for some poor clients, taking the daily pill may only be possible when they forego their comfort and well-being to take their medicines even when there is no food.^6^ Furthermore, avoiding strenuous activity or worries in a context of economic scarcity may be difficult without money. Similar observations are made in studies of PLHIV on ART in Tanzania and Western Kenya.^3,4^ These findings underline the necessity of programs to empower PLHIV on ART in resource-poor settings economically if they are to adhere to the treatment regime. The findings further point to the need to invest in improving the quality of treatment for comorbidities and continuous psychosocial support to minimize patients’ reliance on herbs and encourage them to continue on treatment amid the constraints, respectively.

Realization of a normal and full life by PLHIV on free ART is further complicated by “exceptionalism” in the arrangement and delivery of ART services. Exceptionalism describes the tendency to implement unique responses above and beyond regular health interventions in the management of a disease.^32^ It is identified as a common feature of HIV/AIDS interventions in sub-Saharan Africa and Uganda in particular.^33^ Exceptionalism has both negative and positive consequences.^32^ In Uganda, like elsewhere in the world, AIDS exceptionalism has generated immense resources for HIV treatment.^1^ However, some of the peculiar arrangements such as strict rules on clientship, packaging of drugs, isolated ART clinics, and the open approach to triage may pose constraints that increase the financial and other costs of seeking and continuing on treatment. For instance, our data show that the restrictions on changing treatment centers forced patients who lived in uncertainty over future prospects to cling onto slots at distant facilities at a huge cost. Packaging ARV medicines in special containers increased prospects for stigmatization of PLHIV, while the isolated clinics and open approach to triage compromised patients’ anonymity and privacy, which forced them to incur additional costs to avert the risks. The stigma generated by ART exceptionalism and PLHIV’s tireless efforts to protect their anonymity and privacy and manage anticipated stigma at household and community levels have been discussed elsewhere.^25^ Mechanisms to integrate ART with the regular health services provided at the facilities, improve privacy at clinics, blend the packaging of ARV medicines with that of common medicines, and establish a harmonized information management system to ease tracking of PLHIV across treatment programs may help to minimize exceptionalism and its attendant repercussions.

Our data portray the central role money plays in the realization of a normal and full life by PLHIV even when on free ART. This finding resonates with other studies in sub-Saharan Africa that underscore the role of money and/or identify poverty as a key concern in accessing and sustaining adherence to HIV treatment in the context of expanded access to free ART.^3,7,8,22-24^ However, while most of these studies affirm the imperiling effects of income poverty on adherence to ARV medicines, we show that the lack of monetary resources not only derails PLHIV from realizing adherence and its associated health benefits but also other important values in life like marriage and privacy. We show that possession of money enables PLHIV to surmount endemic structural and health-system barriers to normalization, a process that also involves adherence. With money, PLHIV are able to meet routine transport costs and ensure food security and access to medicines amid stock-outs, which are highlighted as significant barriers to ART adherence in Africa.^6,19,21,22,34^ Using money, clients are able to manage uncertainty and ensure their continuation on treatment. Access to adequate monetary resources further helps to neutralize the effects of unfavorable treatment arrangements and stigma on the attainment/protection of valued goals in life like privacy and dignity and marriage and intimacy. Therefore, it could be argued that in the context of free ART scale-up, financial resources and hence interventions to empower PLHIV economically are critical not merely for facilitating adherence to medicines but the realization of the normal life that ART promises for PLHIV. Russell and Seeley and McGrath et al appear to corroborate this view when they identify economic hardships as key barriers to PLHIV’s realization of a normal life with chronic disease.^11,25^ However, it should be noted that possession of money may not be able to address psychosocial barriers like feelings of guilt, dilemmas of disclosure, the distress of taking medication for life, and personal struggles to comply with behavioral requirements of the ART regime due to addictions and social pressure.^35,36^ Addressing such constraints requires robust psychosocial interventions such as post-ART initiation counseling to enable continuous encouragement, education, and enhancement of the problem-solving capacities of PLHIV on treatment.

The study affirms that structural barriers such as fears of stigma, poverty, and health-system limitations remain key constraints to ART adherence and therefore realizing its primary benefit of normalizing life as shown in previous studies.^3,4,6,28,34^ In addition, this study shows that structural barriers increase the financial and psychosocial costs of normalizing life while on free ART. It is evident that several PLHIV managed to overcome the barriers to adhere to ART, protect privacy and anonymity, and sustain intimate relationships. However, these goals were realized at a considerable monetary, temporal, and psychosocial cost, sometimes at the expense of the patients’ physical and psychological well-being. The economic and psychosocial costs of adhering to ART and protecting anonymity are implicit in several studies in Uganda and other parts of sub-Saharan Africa.^4,6,37,38^ The findings underscore the need for supportive interventions that address the broader contextual issues that impinge on everyday living if PLHIV on medicine are to realize the values that are integral to a normal and full life within their local setting. Such interventions may include economic empowerment programs targeting PLHIV on ART and massive awareness campaigns on the preventive benefits of ART to reduce stigma associated with the infectious nature of HIV.

Unfortunately, the supportive aspects of ART have been given limited attention in Uganda and other sub-Saharan African countries.^28,39,40^ For instance, a report on the performance of Uganda’s HIV/AIDS sector indicates that treatment and care consume more than half (51%) of the resources for HIV/AIDS in the country. Supportive elements including OVC (orphans and other vulnerable children) and social support, social services, and the creation of an enabling environment were in total allocated a meagre 6.2%.^41^ In addition, existing supportive interventions like HIV counseling are shown to be not only poorly resourced^23^ but also mainly focusing on providing information for adherence to medicines and the treatment regime in general.^42^ However, they do not holistically empower clients to take on the intricate psychosocial and economic risks that constrain them from realizing the life that people in their communities aspire to lead.

The study findings may be limited in several ways. First, we focused on PLHIV enrolled at only 2 health facilities in Mukono district. Therefore, the findings may not reflect the experiences of PLHIV on ART in the whole of Mukono, the Central, and other regions of Uganda. However, similar findings have been reported in other parts of the country.^6,13,19,22,25^ Secondly, the study participants were adult PLHIV. Thus, the findings may not apply to ART clients aged below 18 years.

## Conclusions

We have shown that provision of free HIV medicines and clinical care is not sufficient to enable normalization of life for PLHIV living in a context of economic scarcity and stigma. The peculiar demands and arrangements of ART services, HIV-related stigma and discrimination, poverty, and a weak health system combine to necessitate that PLHIV on free HIV treatment continuously pay economically and psychosocially to attain good health and other values that define a normal and full life within their sociocultural settings.

It is thus imperative that free ART is complemented with proactive arrangements that empower PLHIV economically, provide holistic psychosocial support with emphasis on enhancing clients’ individual problem-solving capacities, and create an enabling environment such as through addressing stigma and improving the quality of treatment and privacy at clinics. Such a multipronged approach would enable a more positive person: environment fit, which is necessary if the promise of a normal life that ARV medicines embody, is to become a banal reality for PLHIV in resource-poor settings.

## Supplemental Material

Supplemental Material, Interview_Guide_for_PLHIV - Paying to Normalize Life: Monetary and Psychosocial Costs of Realizing a Normal Life in the Context of Free Antiretroviral Therapy Services in Uganda Click here for additional data file. Supplemental Material, Interview_Guide_for_PLHIV for Paying to Normalize Life: Monetary and Psychosocial Costs of Realizing a Normal Life in the Context of Free Antiretroviral Therapy Services in Uganda by Esther Kalule Nanfuka, David Kyaddondo, Sarah N. Ssali and Narathius Asingwire in Journal of the International Association of Providers of AIDS Care (JIAPAC)

## Notes

1. US$1 = 3700 UGX.

2. In addition to legally registered unions, marriage in the Ugandan local context includes consensual unions of 2 people that are sanctioned by the community as husband and wife.
