# Supplementary material for: Paying to Normalize Life: Monetary and Psychosocial Costs of Realizing a Normal Life in the Context of Free Antiretroviral Therapy Services in Uganda
Source: J Int Assoc Provid AIDS Care. 2019 Jul 3;18:2325958219859654. doi: 10.1177/2325958219859654 (PMC6748487; doi:10.1177/2325958219859654)
Supplement: Supplemental Material, Interview_Guide_for_PLHIV - Paying to Normalize Life: Monetary and Psychosocial Costs of Realizing a Normal Life in the Context of Free Antiretroviral Therapy Services in Uganda [file Interview_Guide_for_PLHIV.pdf]

Research Topic: Mobilisation of resources for managing HIV: Practices of People Living with HIV on antiretroviral therapy in Uganda

Interview guide for People Living with HIV/AIDS

Study Area Identifiers

District

Sub County/Division

Name of Health Unit

Level of unit

Village/Zone

Section 1: Socio demographic characteristics

1. 1 Name

1.2 Age

1.3 Sex

1.4 Marital status

1.5 Highest education level attained

1.6 Residence: District/Sub County/Division/Village/Zone

1.7 Main Source of livelihood

1.8 Ethnicity

1.9 Household size

Section 2: History of HIV/AIDS illness

2.1 Now let us discuss in more detail about how you got to know that you are HIV positive and the steps you took to get enrolled on an HIV/AIDS programme.

Learning about HIV status

- When did you learn about your HIV status?
- How did you get to know that you are HIV positive? In which year did you test? Where did you test from?
- Whom did you disclose your status to? Probe in to the reasons why that particular person (s) were disclosed to and not others? If not disclosed why?

Starting ART

- What HIV related medicines are you taking?
- When did you get enrolled on ART/ other HIV/AIDS programme? Please tell me the journey of how you started on medication? Which people helped you to start ART/

other HIV/AIDS programme? What different places have you received ART from since you started?

- **Tell me about the HIV status of other members of your household.** (Those with HIV and how you are related)
  Those on ART (Is any of your children enrolled on ART or other HIV/AIDS service; is your partner on ART or any other programme? Is any other person in your household enrolled on ART or any other HIV/AIDS service programme?

Section 3: Resources

3.1 Now let us discuss the important things (resources) you need to manage HIV /AIDS. Let us start with the things (resources) you consider important to have in the home.

- Which things do you consider important to manage HIV at home? Please name/list them (probe for treatment supporters, medicines (ARVs and others), information for self-care etc)
- You have listed several resources that are important to manage the illness in the home. Now let us rank them according to their order of importance? What do you consider the most important resource for the management of HIV/AIDS in the home? Why?
- Where are the various resources obtained?
- If you compare yourself (having HIV) and other members of your household without HIV, which things/resources do take to be important for you to have in the home but may not necessarily be important for other household members without HIV? Probe why?
- Which things do you consider important to have in the home now that you are on ART that you did not consider important to have in your home before you started ART.

3.2 Now let us discuss the things/resources you consider important to manage HIV/AIDS at the health facility.

- Which things/resources do you consider important for the management of HIV/AIDS that should be in a health facility? Probe for diagnostic equipment/ medicines, infrastructure, health workers etc
- Lets us rank the things you have listed in order of their importance
- Which things do you consider particularly important in the health facility for people with HIV/AIDS but may not necessarily be important for patients without HIV/AIDS?

3.3 Other than this particular health unit (name the unit), which other health facilities do you seek treatment from? Probe: nature facility, types of services sought from that facility(ies), why?.

3.4 How about in the community where you come from, which things do you consider important for the management of HIV/AIDS?

3.5 Which of the identified resources do you actually mobilise? Which ones have you failed and or often fail to mobilise? Why do you manage to mobilise some and fail to get others?

3.6 Where are the identified resources obtained (probe for the sources of specific resources).

4.0 Strategies of Mobilising Resources

4.1 What steps do you take to obtain the resources you consider necessary for the management of HIV at home?

- Probe for medicines (ARVS, other medicines) and other resources at home, health facility and community

4.2 How do relatives, friends, fellow patients, neighbours and other people you know support you to mobilise the necessary treatment resources?

4.3 Who of the people in your social network do you rely on most or is particularly helpful in issues involving mobilisation of resources for the management of HIV? Why do you rely on that particular person? Why do you think they are always willing to support you?

- Probe for support during events of illnesses and when there are no illnesses

4.4 Which constraints do you face in the process of mobilising the necessary resources? Which one(s) do you consider the biggest constraints?

4.5 How do you overcome them to access resources?

4.6 In your opinion which opportunities are there to address these constraints? Probe for opportunities at home, community and health facility levels

Section 5: Health Problems

5.1 Now let us talk about your general health. What diseases do you commonly suffer from?

Probe Qns

- Reasons why you think they are frequent.
- How often do they occur (note occurrence for particular illnesses)?
- Which illnesses are you more scared of? And Why.

Section 6: Treatment and Management of HIV/AIDS related illnesses

6.1 Which illnesses do you often manage at home? Which ones do you commonly seek treatment from a health facility? Which ones do you take to other healers (not in the health facility)?

- How do you choose which illnesses to manage at home, take to the health facility or other healer?

6.2 For the illnesses you often manage at home, what are the most important things/resources you need to manage the illnesses? Would you please tell me different illnesses you often manage at home and the different requirements for their management? (name the illness identified and probe for the resources required).

6.3 You have mentioned several important things/resources. Of these, which ones do you always stock in the home? (Probe for medicines, other pharmaceuticals and people) Where do you obtain them from? Tell me the steps you take to access them?

- Which ones do you obtain only in the event of an illness? What steps do you take to access them?

- How do you choose which things you can stock and those you can only get in the event of illness?

Do you have any comments or questions on the things we have been discussing?

The interview ends here for now. I might contact you for other future discussions. Thank you very much for your time.
